# Construction and validation of prognostic models in critically Ill patients with sepsis-associated acute kidney injury: interpretable machine learning approach

**DOI:** 10.1186/s12967-023-04205-4

**Published:** 2023-06-22

**Authors:** Zhiyan Fan, Jiamei Jiang, Chen Xiao, Youlei Chen, Quan Xia, Juan Wang, Mengjuan Fang, Zesheng Wu, Fanghui Chen

**Affiliations:** 1 Department of Emergency, Hangzhou First People’s Hospital Affiliated to Zhejiang University School of Medicine, 310006 Hangzhou, Zhejiang China; 2 Department of Ultrasound, The First Affiliated Hospital Zhejiang University School of Medicine, 310003 Hangzhou, Zhejiang China

**Keywords:** Acute kidney injury, Sepsis, Mortality, Critical illness, MIMIC-IV database, Prognosis, Machine learning, SHAP

## Abstract

**Background:**

Acute kidney injury (AKI) is a common complication in critically ill patients with sepsis and is often associated with a poor prognosis. We aimed to construct and validate an interpretable prognostic prediction model for patients with sepsis-associated AKI (S-AKI) using machine learning (ML) methods.

**Methods:**

Data on the training cohort were collected from the Medical Information Mart for Intensive Care IV database version 2.2 to build the model, and data of patients were extracted from Hangzhou First People's Hospital Affiliated to Zhejiang University School of Medicine for external validation of model. Predictors of mortality were identified using Recursive Feature Elimination (RFE). Then, random forest, extreme gradient boosting (XGBoost), multilayer perceptron classifier, support vector classifier, and logistic regression were used to establish a prognosis prediction model for 7, 14, and 28 days after intensive care unit (ICU) admission, respectively. Prediction performance was assessed using the receiver operating characteristic (ROC) curve and decision curve analysis (DCA). SHapley Additive exPlanations (SHAP) were used to interpret the ML models.

**Results:**

In total, 2599 patients with S-AKI were included in the analysis. Forty variables were selected for the model development. According to the areas under the ROC curve (AUC) and DCA results for the training cohort, XGBoost model exhibited excellent performance with F1 Score of 0.847, 0.715, 0.765 and AUC (95% CI) of 0.91 (0.90, 0.92), 0.78 (0.76, 0.80), and 0.83 (0.81, 0.85) in 7 days, 14 days and 28 days group, respectively. It also demonstrated excellent discrimination in the external validation cohort. Its AUC (95% CI) was 0.81 (0.79, 0.83), 0.75 (0.73, 0.77), 0.79 (0.77, 0.81) in 7 days, 14 days and 28 days group, respectively. SHAP-based summary plot and force plot were used to interpret the XGBoost model globally and locally.

**Conclusions:**

ML is a reliable tool for predicting the prognosis of patients with S-AKI. SHAP methods were used to explain intrinsic information of the XGBoost model, which may prove clinically useful and help clinicians tailor precise management.

**Supplementary Information:**

The online version contains supplementary material available at 10.1186/s12967-023-04205-4.

## Background

Sepsis-associated acute kidney injury (S-AKI) is one of the most common diseases in hospitalized and critically ill patients; it is not only associated with an increased risk of chronic kidney disease but also with high morbidity and mortality rates [1–4]. Little is known about the epidemiology of S-AKI. Sepsis causes over 5.3 million deaths annually, with approximately 30% overall mortality, especially in the ICU [5, 6]. Extrapolating from the incidence rate in the United States, Adhikari et al. estimated 19 million sepsis cases worldwide per year. However, the true incidence rate is presumably much higher. As approximately one in three patients with sepsis will develop AKI [7], the annual global incidence of S-AKI may be approximately 6 million. Nevertheless, this number is lower than the estimates extrapolated from AKI incidence. The development of AKI in patients with sepsis is associated with increased mortality [8], resulting in a heavy burden on both patients and society.

However, the pathophysiological mechanisms underlying S-AKI remain poorly understood. What is certain is that these mechanisms are consistent with the organ injury associated with sepsis, including inflammation, microcirculatory dysfunction, and metabolic reprogramming.

Considering the high incidence and mortality rates, it is necessary to establish a reliable and efficient prognostic model for S-AKI. Several risk prediction models for AKI in critically ill patients have been widely studied and established [9–11]. da Hora Passos has developed a clinical score to predict early mortality in S-AKI, which merely centered on patients treated with continuous renal replacement therapy (CRRT) rather than critically ill patients, with a small sample size and lack of external validity [12]. Furthermore, the application of general severity scores in specific cohorts is controversial because of relatively unsatisfactory discrimination and calibration. Ohnuma demonstrated that most part of AKI-assessed scores published in the twenty-first century included general severity scores with a significantly low calibration ability [13]. Recently, Hu et al. proposed and validated a specific clinical model to predict the survival of critically ill patients with S-AKI [14]. However, the prediction model was based on traditional COX regression.

In recent years, diverse machine learning (ML) algorithms, a data analysis method that develops algorithms to forecast outcomes by “learning" from data, have been examined for the early revelation of S-AKI and it outperformed the traditional statistical methods, which require no assumptions regarding input variables and their relationships with the output. The advantage of completely data-driven learning without reliance on rules-based programming is that ML constitutes a reasonable approach. Tseng et al. [15] revealed that a prediction model established by ML techniques confirmed risk factors following cardiac surgery, which enabled the optimization of postoperative interventions to reduce the postoperative complications following cardiac surgery. Dong et al. [16] developed an ML model to learn predisease patterns of physiological measurements and predict pediatric AKI up to 48 h in advance compared with presently established diagnostic guidelines. Furthermore, Zhang et al. [17] demonstrated that the XGBoost model could separate and sort patients into those who would and would not respond to fluid intake in the urine output better than the traditional logistic regression model. Yue [18] has developed an ML model for the early identification of critically ill patients with S-AKI and showed that the XGBoost model had the best predictive performance, which can be used to assist clinicians in identifying high-risk patients to minimize the mortality. These studies above suggest that ML algorithms can improve the development and validation of prediction models in critical care research. However, the primary outcome of all studies mentioned above was AKI detection rather than poor clinical outcomes, such as mortality due to AKI. Therefore, we aimed to develop a prognostic prediction model based on ML in critically ill patients with S-AKI. Furthermore, despite the promising performance of ML algorithms in previous studies, it is difficult to explain what features of the patient are responsible for the given prediction, owing to the "black-box” nature of ML algorithms. To date, the lack of interpretability has been a major obstacle to the implementation of ML models in the medical field [19]. To interpret the results of ML models, we combined an advanced ML algorithm with a method based on SHapley Additive exPlanations (SHAP). SHAP is a popular ML technique for obtaining insights into the complicated relationships between characteristics and predictions [20]. In addition to optimizing the predictive performance of mortality risk in critically ill patients with S-AKI, this study provides intuitive explanations that will help clinicians comprehensively understand the process of how the developed model makes a particular prediction and increase the opportunity for early interventions.

Accordingly, the purpose of this study was twofold: first, we aimed to determine the best-performing ML models in the prediction of short-term mortality in S-AKI patients; second, we planned to use an interpretable ML, by combining the SHAP value to examine risk factors and quantitatively visualize the relationships between risk factors and outcomes.

## Methods

### Training cohort

An open and free critical care database called the Medical Information Mart for Intensive Care IV database (MIMIC-IV) version 2.2 [21–23], which is the latest version that contains comprehensive clinical data of patients admitted to the Beth Israel Deaconess Medical Center between 2008 and 2019. MIMIC-IV, an update of the MIMIC-III, incorporates contemporary data and improves numerous aspects of MIMIC-III. It catalogs > 200,000 emergency department admissions and  > 70,000 ICU stays. The clinical data in the database consist of demographic characteristics, vital signs, imaging examinations, laboratory test results, data dictionary, and documents containing codes of the International Classification of Diseases, Ninth and Tenth Revisions (ICD-9 and ICD-10, respectively) and records of hourly physiologic data from beside monitors validated by ICU nurses; The health information obtained from the MIMIC-IV database was unidentified, so informed consent of patients was not required [21, 24]. An author (ZY Fan) was approved to extract data from the database for research purposes (Certification No. 46451755). This database was approved by the Institutional Review Boards (IRB) of the Massachusetts Institute of Technology (MIT).

Sepsis is defined as a life-threatening organ dysfunction caused by a dysregulated host response to infection (sepsis 3.0) [5]. Organ dysfunction may be identified as an acute and infection-related change of at least two points in the sequential organ failure assessment (SOFA) score.

AKI is identified and sorted by the basis of the highest serum creatinine (SCr) level and urine output as stated by Kidney Disease Improving Global Outcomes (KDIGO) [25]. Definition as follows: increase in SCr to ≥ 1.5 times baseline must have occurred within the prior 7 days; or a ≥ 0.3 mg/dL increase in SCr occurred within 48 h; or urine output < 0.5 mL/kg/h for 6 h or more. If the preadmission SCr was not recorded, the first SCr value at admission was used as the baseline SCr. In this study, AKI was evaluated by the worst serum creatinine and urine volume within 72 h after the suspected diagnosis of sepsis.

### External validation cohort

Patients were enrolled from Hangzhou First People’s Hospital Affiliated to Zhejiang University School of Medicine (Zhejiang, China) between 2018 and 2022. Adult patients who had a diagnosis of S-AKI were included. The exclusion conditions were same as training cohort. This study was reviewed and approved by the Ethics Committee of Hangzhou First People’s Hospital Affiliated to Zhejiang University School of Medicine (KY2022124).

### Data extraction

**Fig. 1:**
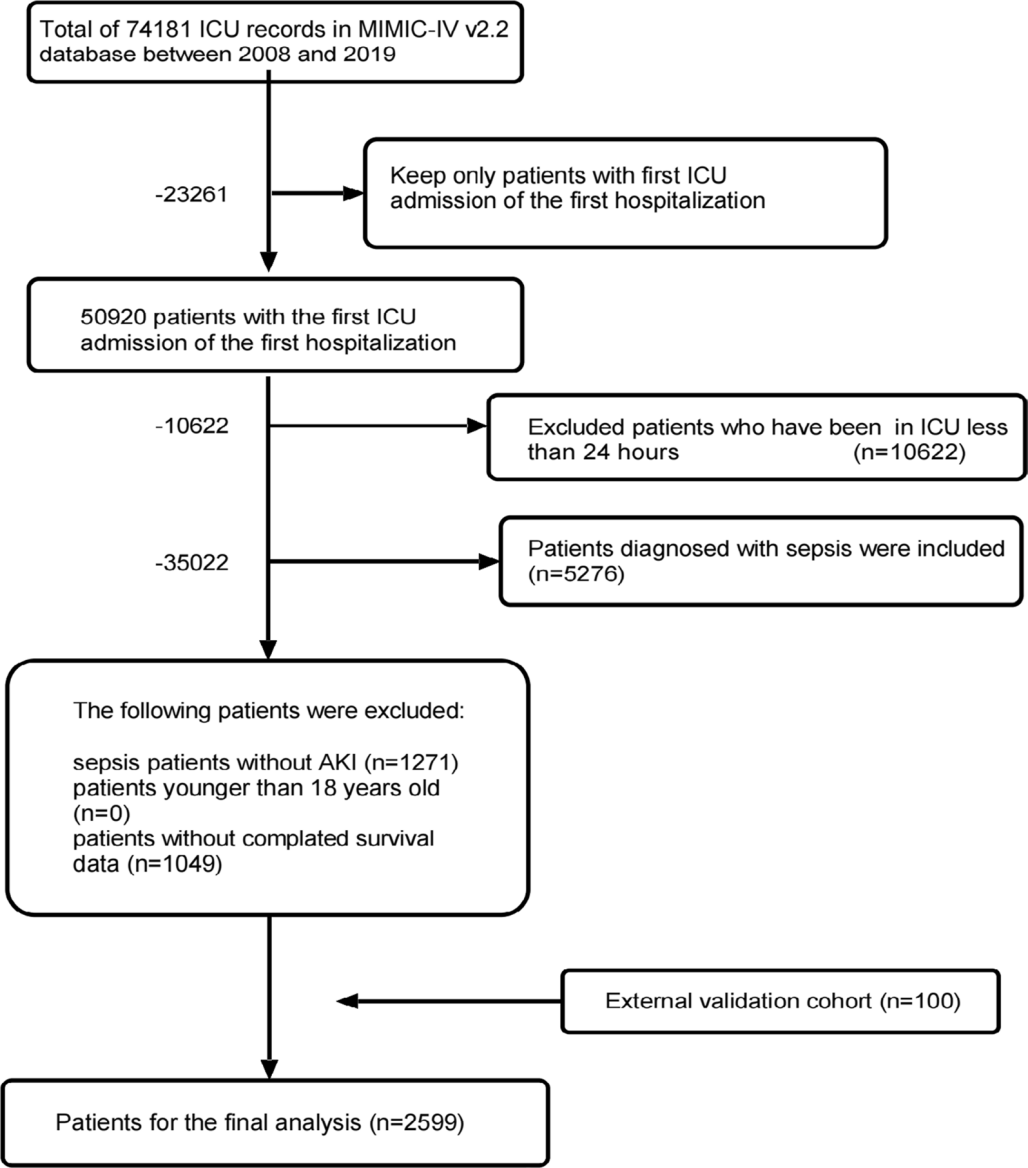
Flowchart of screening

We first obtained raw data using Structured Query Language with Navicat Premium software (version 15.0.12). Structured Query Language was used to extract patient data, including sociodemographic characteristics, vital signs, laboratory parameters, complications, and microbiological information [26]. Patients in the database who met the following criteria were selected for the present study: [1] first ICU admission at first hospitalization; [2] ICU length of stay > 24 h; [3] age of > 18 years; [4] and met the diagnostic criteria for sepsis 3.0 and AKI development according to the criteria. ICD-9 (99591, 99592, and 78552) and ICD-10 (R65.20, R65.21) codes were used to identify patients with sepsis in the MIMIC-IV database. Data of these patients were used as the training cohort for model establishment. The data extraction procedure is illustrated in Fig. 1.

We extracted the following demographic data: age at admission, sex, ethnicity, weight, height, length of stay in the ICU, and hospital expire flag (the recording of in-hospital death in the database) at the first ICU admission. Next, the vital signs of the patients in the first 24 h of ICU stay, including mean arterial pressure (Meanbp), heart rate, temperature, respiratory rate, oxyhemoglobin saturation (SpO_2_), urine output and then laboratory parameters in the first 24 h, including routine blood examination, liver and kidney function, blood glucose, and arterial blood gas (ABG), were collected. In addition, advanced life support recordings, such as mechanical ventilation and renal replacement therapy, were recorded. Comorbidities were identified using the Charlson table in materialized view.

**Fig. 2:**
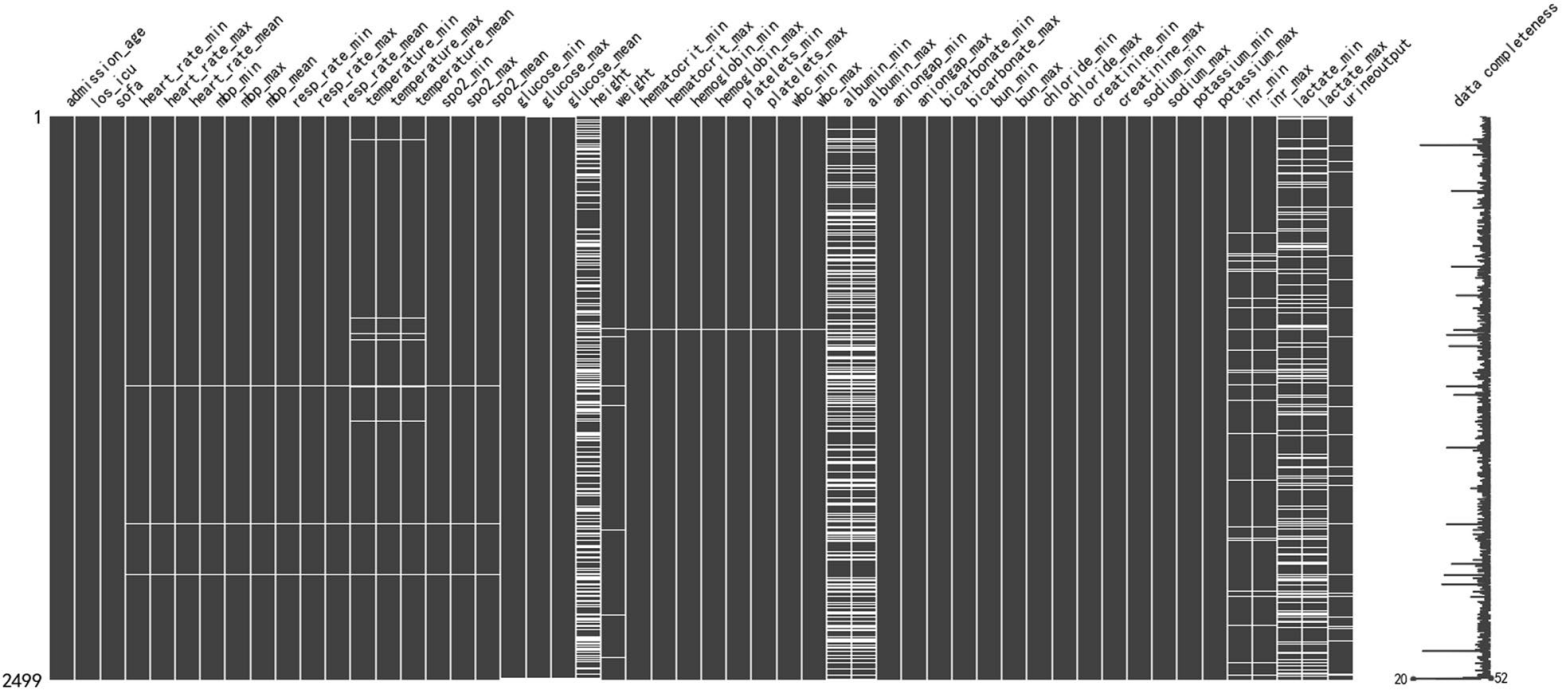
Missing data distribution each column represents a clinical variable and the white line represents the missing data. The more withe lines in each column, the more missing values for that variable

To filter for missing data, the missingno module in Python 3.9.12 software was used. In Fig. 2, each column represents a clinical variable, and the white line represents the missing data. The denser withe lines in each column, the more missing values there are for that variable. Detailed information regarding missing values is provided in Additional File 1. We removed variables missing > 30% of observations, such as height and serum albumin levels, to facilitate and ensure study accuracy. Missing values were imputed using multivariate imputation by chained equations [27]. The maximum, minimum, and mean values were used when incorporating the characteristics of vital signs and related laboratory parameters and were considered as independent features to be included in the study.

### Statistical analyses

Normality testing was performed by Shapiro-Wilks test. Continuous variables with normal distributions are presented as the mean (SD, standard deviation) and compared with independent samples *t* tests. Non-normally distributed variables are expressed as the median (interquartile ranges), which were compared with Kruskal–Wallis test. Categorical variables were described as percentages and were compared using the chi-square test. Patients were categorized into “survival” and “non-survival” groups, according to their survival status within 7, 14, or 28 days. Specifically, these were 7-day survival and non-survival groups, 14-day survival and non-survival groups, and 28-day survival and non-survival groups. Variables are displayed and compared in groups of 7 days in Table 1.

**Table 1:** The characteristics of training cohort patients when first ICU admission

Variables	Survival (n=1787)	Non-survival(n=712)	P-value
Age (year)	72 (62, 83)	75 (63, 84)	0.0565
Gender (%)			
Female	773 (43.3)	337 (47.3)	0.0675
Male	831 (57.3)	253 (58.4)	
Ethnicity (%)			
White	1209 (67.7)	470 (66.0)	<0.001
Black	176 (9.8)	42 (5.9)	
Yellow	60 (3.4)	22 (3.1)	
Others	342 (19.1)	178 (25.0)	
Weight (kg)	78.0 (64.9, 95.9)	76.8 (63.4, 94.0)	0.13
Admission type (%)			
Elective	191 (10.7)	75 (10.5)	0.722
Emergency	1095 (61.3)	426 (59.8)	
Urgent	501 (28.0)	211 (29.6)	
First Care unit (%)			
MICU	739 (41.3)	299 (42)	0.213
SICU	706 (39.5)	284 (39.8)	
CCU	80 (11.9)	64 (9.0)	
TSICU	161 (9.0)	49 (6.9)	
Others	101 (5.7)	16 (2.2)	
SOFA	9 (6, 12)	11.0 (8, 14)	<0.001
AKI stage (%)			
I	227 (12.7)	46 (6.5)	<0.001
II	627 (35.1)	158 (22.2)	
III	933 (52.2)	508 (71.3)	
Length of ICU stay (day)	6.54 (3.0, 12.1)	2.86 (1.8, 4.6)	<0.001
Comorbidity			
Congestive heart failure, n (%)	a720 (40.3)	242 (34.0)	0.00356
Chronic pulmonary disease, n (%)	a229 (12.8)	83 (11.7)	0.461
Diabetes complicated, n (%)	227 (12.7)	64 (9.0)	0.00862
Renal disease, n (%)	574 (32.1)	182 (14.8)	0.00123
Liver disease, n (%)	452 (25.3)	226 (31.7)	0.0012
Solid tumor, n (%)	385 (21.5)	179 (25.1)	0.0563
Metastatic cancer, n (%)	187 (10.5)	132 (18.5)	<0.001
AIDS, n (%)	16 (0.9)	4 (0.6)	
Vital signs^a^			
Heartrate_mean (min^-1^,mean SD)	90.5 ± 17.2	96.2 ± 18.1	<0.001
Heartrate_max(min^-1^,mean SD)	112 ± 23.1	117 ± 23.3	<0.001
Meanbp_mean (mmHg)	71.9 (66.9, 77.3)	69.9 (64.7, 75.3)	<0.001
Meanbp_min (mmHg)	53 (47, 59)	50.0 (41, 59)	<0.001
Resprate_mean (min^-1^)	20.4 (17.7, 23.7)	22.4 (19.4, 25.8)	<0.001
Resprate_max(min^-1^)	29.0 (25, 34)	31.0 (27, 36)	<0.001
Temperture_mean (℃)	36.8 (36.6, 37.2)	36.7 (36.4, 37.2)	0.809
Temperture_max (℃)	37.3 (37, 38)	37.2 (36.8, 38)	0.0148
SpO_2__mean (%)	97.0 (95.6, 98.4)	96.2 (94.4, 97.8)	<0.001
SpO_2__min (%)	92.0 (89, 94)	90.0 (86, 93)	<0.001
Urine output (mL)	1000 (556.5, 1625)	579 (223.8, 1102)	<0.001
Laboratory parameters^b^			
Aniongap_max (mmol/L)	18.0 (15, 21)	20.0 (17, 24)	<0.001
Bicarbonate_min (mmol/L)	19.0 (16, 23)	17.0 (13, 20)	<0.001
Lactate_max (mmol/L)	2.4 (1.6, 4.2)	3.7 (2.2, 7)	<0.001
Glucose_mean (mg/dL)	135 (111.4, 170.7)	135 (104.5, 189.0)	0.804
Creatinine_max (mg/dL)	1.70 (1.1, 2.7)	2.0 (1.3, 3.1)	<0.001
Chloride_min (mmol/L)	101 (97, 106)	100 (96, 106)	0.0266
Chloride_max (mmol/L)	106 (101, 111)	106 (100, 111)	0.569
Hematocrit_min (%)	28.3 (24.2, 33)	28.5 (23.9, 33.4)	0.987
Hemoglobin_min (g/dL)	9.20 (7.9, 10.7)	9.15 (7.7, 10.8)	0.459
Platelet_min (10^9^/L)	159 (98.5, 234)	138 (68, 213.25)	<0.001
Potassium_min (mmol/L)	3.80 (3.5, 4.3)	4.0 (3.5, 4.5)	<0.001
Potassium_max (mmol/L)	4.50 (4.1, 5.2)	4.80 (4.2, 5.5)	<0.001
Sodium_max (mmol/L)	140 (136, 143)	140 (136, 143)	0.837
INR_min	1.40 (1.2, 1.7)	1.50(1.2, 1.9)	<0.001
INR_max	1.50 (1.3, 2.1)	1.80 (1.4, 2.7)	<0.001
BUN_max (mmol/L)	29.0 (22, 56)	34.0 (28, 64)	<0.001
WBC_max (10^9^/L)	35.0 (10.5, 21.9)	42.0 (10.3, 23.7)	<0.001
Advanced life support			
Renal replacement therapy (%)	125 (7.0)	70 (9.8)	0.0205
Mechanical ventilation (%)	1058 (59.2)	492 (69.1)	<0.001
Sources of infection, n (%)			
Blood	50 (2.8)	55 (7.7)	<0.001
Urine	201 (11.2)	55 (7.7)	0.00845
Sputum	196 (11.0)	28 (3.9)	<0.001
MRSA screen	36 (2.0)	9 (1.3)	0.245
Other	52 (2.9)	11 (1.5)	0.0587

Categorical data were showed as frequency (percentage). Continuous variables with normal distributions were presented as the mean (SD, standard deviation) and compared with independent samples t tests. Non-normally distributed variables are expressed as the median (interquartile ranges), which were compared with Kruskal-Wallis test

^a^Vital signs data were calculated as mean/minimum/maximum value during the first 24h of ICU admission

^b^The laboratory parameters recorded the mean/minimum/maximum value during the first 24h since ICU admission of each included patients

*SD* standard deviation, *CCU* coronary care unit, *MICU* medical intensive care unit, *SICU* surgical intensive care unit, *TSICU* trauma/surgical intensive care unit, *SOFA* sequential organ failure assessment, *AIDS* Acquired Immune Deficiency Syndrome, Meanbp mean arterial pressure, *SpO*_*2*_ oxyhemoglobin saturation, *INR* international normalized ratio, *BUN* blood urea nitrogen, *WBC* white blood cell, *MRSA* methicillin-resistant Staphylococcus aureus

For ML models, scikit-learn Python library (version 1.2.1) and XGBoost (version 1.7.3) packages were used to create models and tune the hyperparameters in Python. During the model-building stage, the “MinMaxScaler” method in the module of “sklearn.preprocessing” was used to scale the data of continuous variables, whereas “OneHotEncoder” method in the module of “sklearn.preprocessing” was used to encode the data of categorical variables. We randomly divided the training cohort patients and allocated 80% to the training set and 20% to the internal validation cohort. The training set was pretreated using the synthesizing minority oversampling technology (SMOTE) with the Tomek link (SMOTETomek) technique to balance positive and negative categories [28]. A recursive feature elimination (RFE) algorithm was used for the feature selection. The ML algorithms considered in this study included random forest (RF), support vector classifier (SVC), logistic regression (LR), XGBoost, and multilayer perceptron classifier (MLP). These were used to construct prediction models. Hyperparameter optimization and cross-validation through GridSearchCV were applied to prevent overfitting and increase model accuracy.

XGBoost is a tree ensemble technique based on the loss generated by weak decision tree-based learners. XGBoost was trained as the baseline model, followed by the training of the final model with optimized hyperparameters. The XGBoost model hyperparameters were tuned using the Scikit-learn GridSearchCV with tenfold cross-validation. The hyperparameters chosen for optimization were learning_rate, gamma, max_depth, subsample, min_child_weight, and n_estimators. The GridSearchCV method of scikit-learn with tenfold cross-validation also tunes the hyperparameters of the SVC, RF, MLP, and LR.

The prediction performance of the five models were assessed by ROC curves and DCA. What’s more, the accuracy, precision, recall, and F1 score of models were also evaluated. The external validation cohort was used to validate the performance of the five models mentioned above by same ways.

SHAP is a flexible method which can be used to explain individual predictions and for global interpretation. It has a substantial theoretical foundation in game theory and uses the concept of allocating optimal credits based on Shapley values to estimate the importance of features. SHAP force plots provide an intuitive visualization of how different features affect an individual prediction. One advantage of SHAP for global interpretation is that SHAP not only reveals about the importance of features but also their relationship with the output. Additionally, SHAP’s predictions are reasonably distributed among feature values. These factors are crucial in guaranteeing trust in the technique [29]. In our work, SHAP feature importance assessment were used for global interpretation of the developed baseline model (Fig. 6). SHAP was also used to come up with examples on how individual predictions can be explained locally (Fig. 7).

## Results

A total of 2599 patients with S-AKI were included in this study, with 2499 included in the training cohort and 100 in the external validation cohort. Patients were categorized into “survival” and “non-survival” groups, according to their survival status within 7, 14, or 28 days. Variables are displayed and compared in groups of 7 days in Table 1.

### Baseline characteristics

Table 1 shows the overall baseline characteristics, vital signs, and laboratory parameters of the training cohort based on the 7-day group. The overall mortality of patients with S-AKI within 7 days was 28% (n = 712) in the training cohort. In univariate analysis, age at admission; AKI stage; SOFA score; comorbidities such as congestive heart failure, diabetes complicated, and metastatic cancer; vital signs such as heart rate, respiratory rate, and SpO_2_; indicators of ABG such as lactate_max, aniongap_max, and bicarbonate_max; blood routine indicators such as platelet count, white blood cell count, serum potassium level, mechanical ventilation, and positive blood culture and sputum culture were considered significant between the groups.

### Features selected in models

This study used the RFE algorithm to select features from the data of training cohort. According to a specific feature ranking standard, RFE starts from a complete set and then eliminates the least relevant feature one by one to select the most important features. Finally, the top of 40 important features were selected by RFE in the three groups, respectively. The order of feature importance was showed in Fig. 6 with SHAP method.

### Model comparison

In the model development and validation stage, we first determined optimal hyperparameters of the XGBoost model for the 7-day group: learning_rate = 0.6, gamma = 0.9, max_depth = 3, subsample = 0.799, min_child_weight = 1, and n_estimators = 2000. The optimal hyperparameters of the XGBoost model for the 14-day group were learning_rate = 0.5, gamma = 0.6, max_depth = 3, subsample = 0.7, min_child_weight = 1, and n_estimators = 2000 and those for the 28-day group were learning_rate = 0.1, gamma = 0.1, max_depth = 5, subsample = 0.799, min_child_weight = 1, and n_estimators = 2000. Detailed information regarding hyperparameters of other ML models is provided in Additional File 2. The final models were trained using optimized hyperparameters.

**Fig 3:**
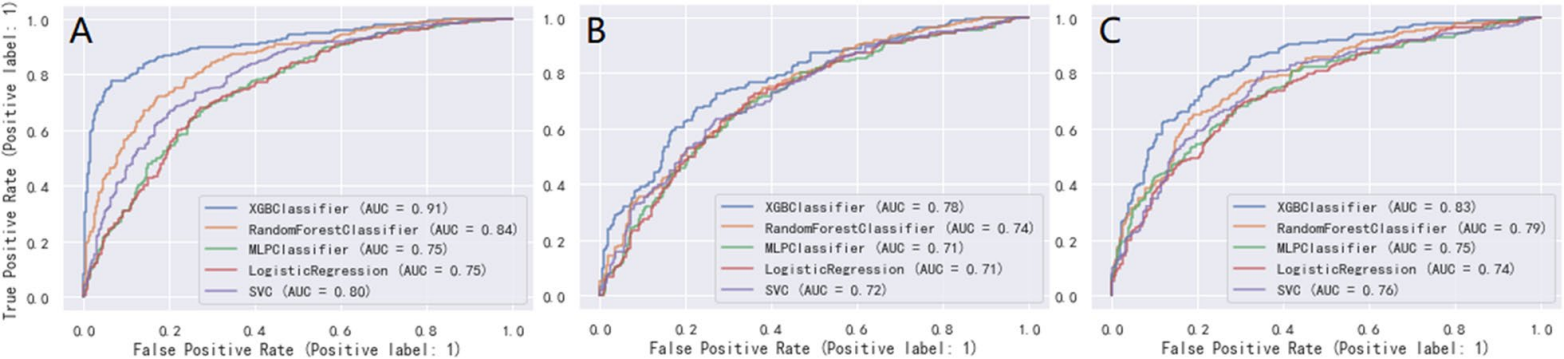
The ROC curves comparison of the five models with the three groups in training cohort. blue line = XGBoost model, organ line = random forest classifier model, green line = multilayer perceptron classifier model, red line = logistic regression model, purple line = support vector classifier model;** A**: 7-day group, **B**: 14-day group, **C**: 28-day group

**Fig 4:**
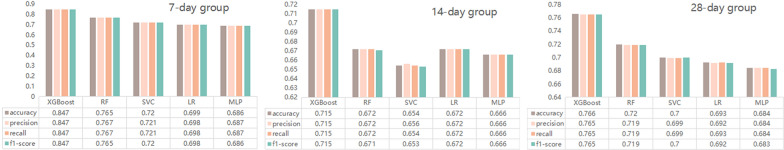
The comparison of performance in the five models with the three groups in training cohort. RF, Random forest model; XGBoost, Extreme Gradient Boosting; MLP, Multi-layer Perceptron classifier; SVC, Support vector Classifier; LR, Logistic regression

**Fig 5:**
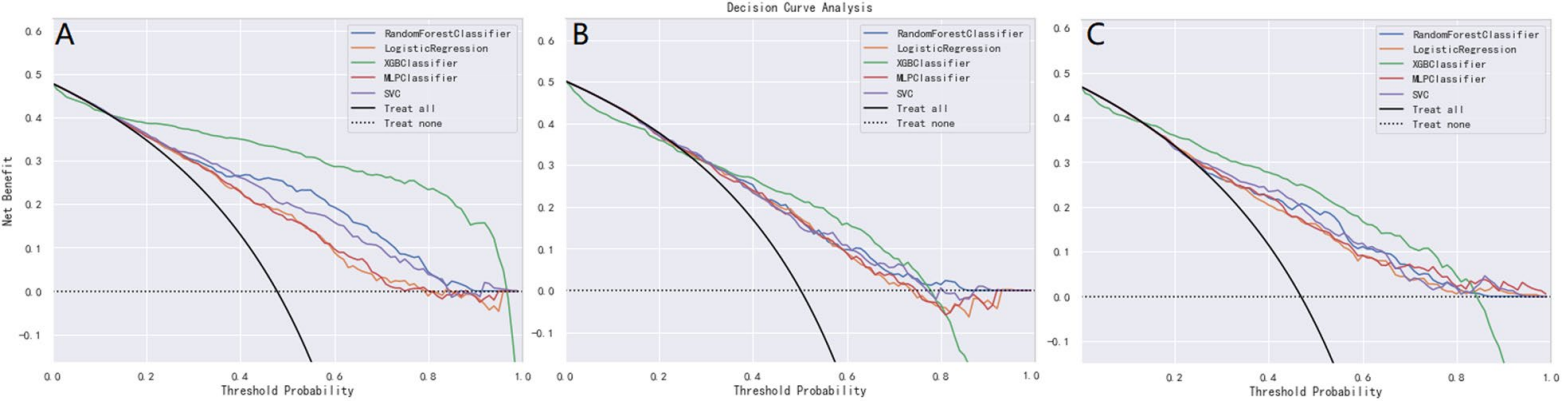
The DCA comparison of the five models with the three groups in training cohort.        Decision curve analysis (DCA) of the five prediction models. The net benefit curves for the prognostic models are shown. X-axis indicates the threshold probability for critical care outcome and Y-axis indicates the net benefit. Green line = XGBoost model, blue line = random forest model, organ line = logistic regression model, red line = multilayer perceptron model, purple line = support vector classifier model;** A**: 7-day group, **B**: 14-day group, **C**: 28-day group

The five ML models (LR, RF, XGBoost, MLP and SVC) demonstrated good discriminative power with AUCs (95%CI) of 0.75 (0.73, 0.77), 0.84 (0.82, 0.86), 0.91 (0.90, 0.92), 0.75 (0.73, 0.77), 0.80 (0.78, 0.82) in the 7-day group, 0.71 (0.69, 0.73), 0.74 (0.72, 0.76), 0.78 (0.76, 0.80), 0.71 (0.69, 0.73), 0.72 (0.70, 0.74) in the 14-day group, and 0.74 (0.72, 0.76), 0.79 (0.77, 0.81), 0.83 (0.81, 0.85), 0.75 (0.73, 0.77), 0.76 (0.74, 0.78) in the 28-day group, respectively. ROC curve comparisons of the five models with the three groups in the training cohort are shown in Fig. 3. The XGBoost algorithm model showed the highest AUC in the 7-, 14-, and 28-day groups. Performance of the RF model was second only to XGBoost, and significantly better than that of the other three models. Results of the F1-score, accuracy, precision and recall of the three groups are shown in Fig. 4. Performance of the XGBoost classification model was better than that of the others in the three groups. According to the DCA results of the five prediction models (Fig. 5), the net benefit of XGBoost was significantly larger than that of the other models in all three groups.

### Interpretability analysis

**Fig 6:**
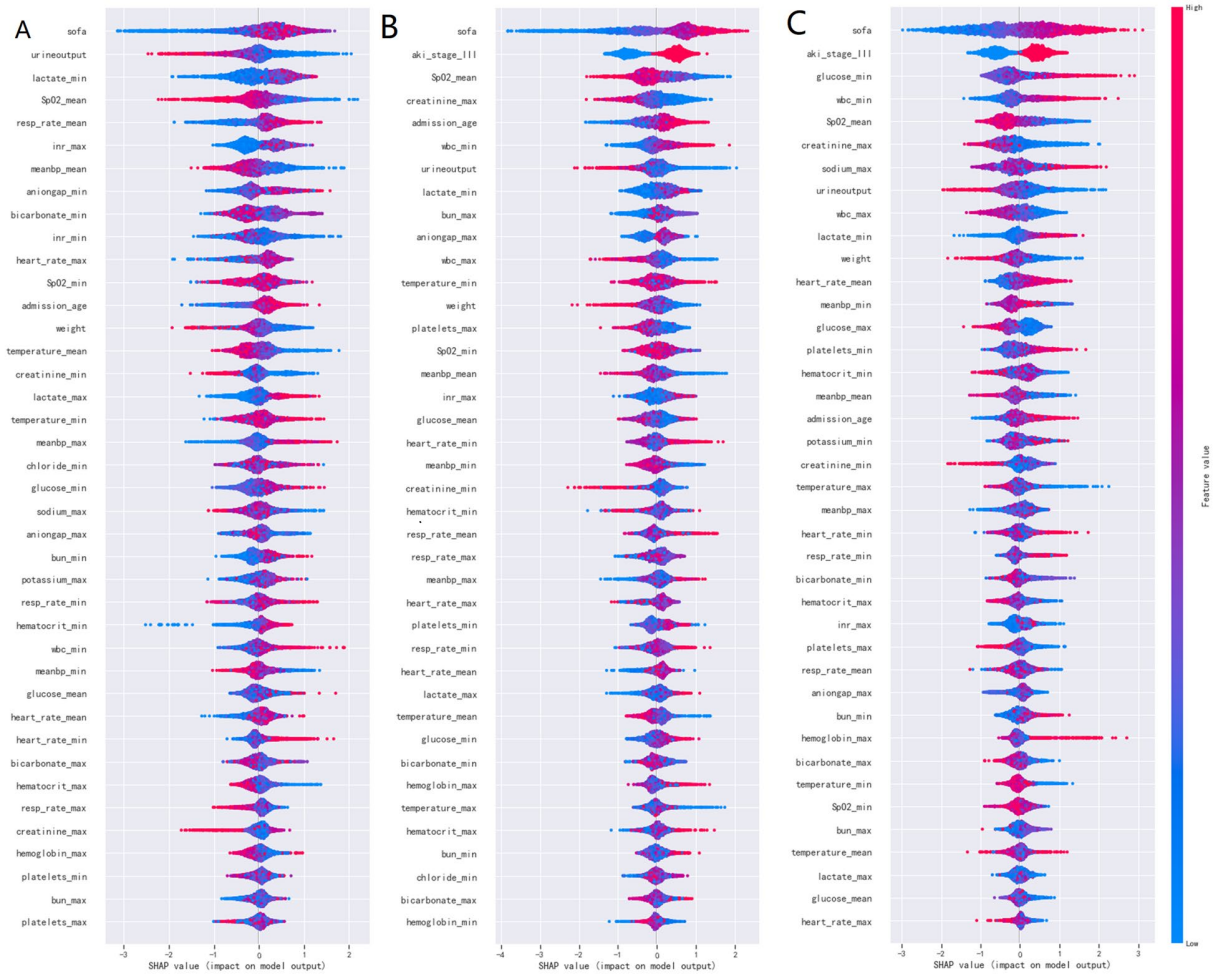
Feature importance analysis by SHAP method for XGBoost model SHAP summary plot of the 40 features of the XGBoost model. A dot is created for each feature attribution value for the model of each patient, and thus one patient is allocated one dot on the line for each feature. Dots are colored according to the values of features for the respective patient and accumulate vertically to depict density. Red represents a high feature value (in this case death), whereas blue represents a low feature value. The further away a point is from the baseline SHAP value of zero, the stronger it effects the output

Initially, the global interpretability of baseline model was studied. The XGBoost model was regarded as the baseline model as it was found to be the best performing model. The feature importance estimates were based on overall samples of training cohort. The global importance of each feature we estimated in SHAP was used to understand the general impact of various features across all samples (see Fig. 6). The summary plot showed all of the 40 features in 7-, 14-, and 28- day groups.

The SHAP summary plot illustrated the entire distribution of each feature’s impact on the model output. The color allowed us to understand how changes in the value of a feature affected the change in outcome. Red represents a high feature value, whereas blue represents a low feature value. The further away a point is from the baseline SHAP value of zero, the stronger it effects the output. This way a features relationship with the SHAP value (and in turn the predicted output) can be better understood. In these three groups, the patient's SOFA score when admitted to the ICU, indicators reflecting circulatory dysfunction (meanbp_max, lactate_min, and urine output), the mean value of SpO_2_ within the first 24 h after ICU admission, AKI stage played a crucial role compared with the other risk factors (e.g., platelets, hematocrit, hemoglobin) of which the distributions of SHAP values by and large were crowded in the center. The direction of effects revealed that high SOFA with a long-right tail led to a high risk of death, whilst high urine output with a long-left tail were also significant and inversely related to predicted death.

**Fig 7:**
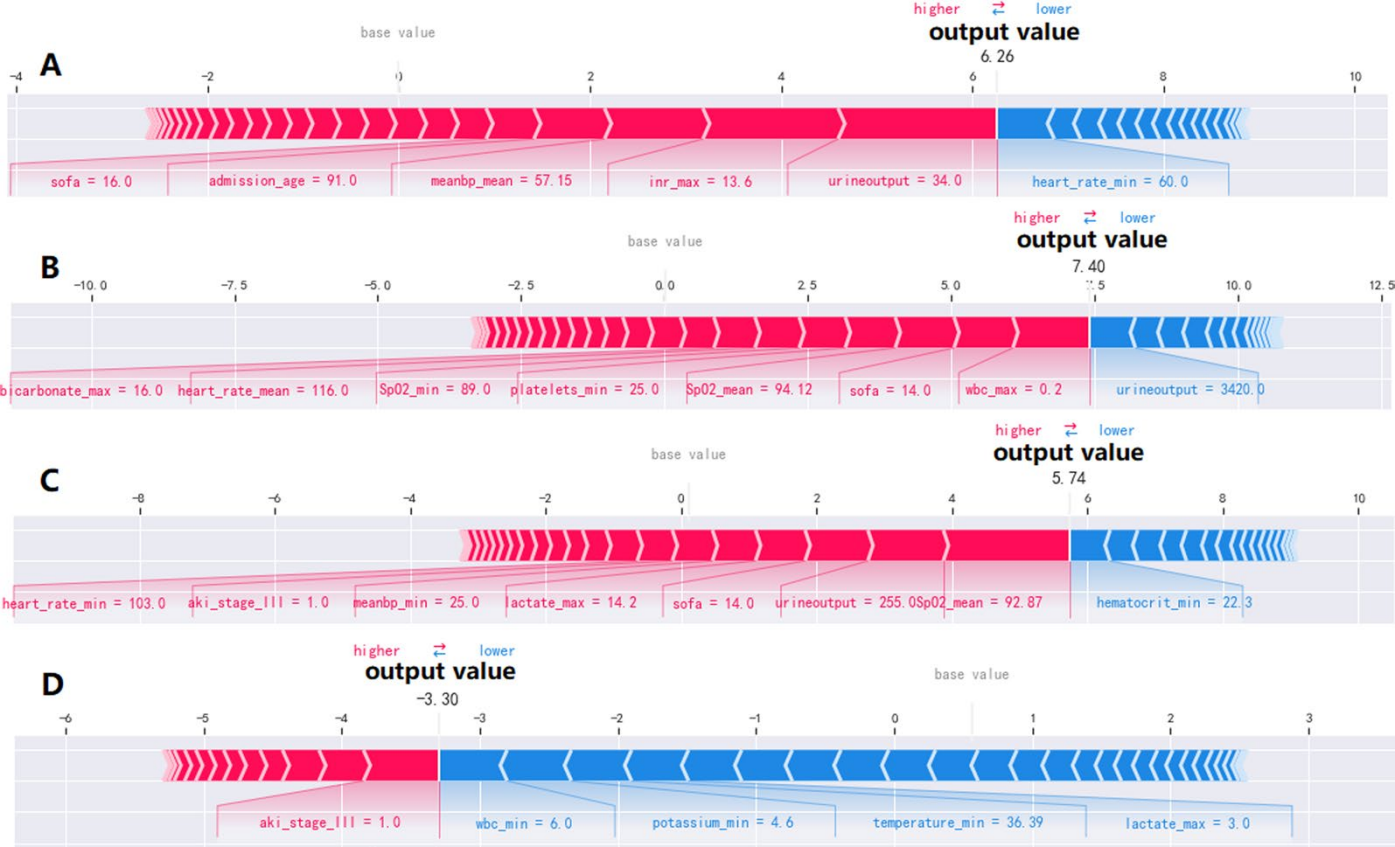
Force plots visualized individual model prediction as result of feature contributions. The base value represented the averaged predicted results. Feature values were listed at the plot bottom with feature names. Each group of features was ranked from center to both ends by the extent of their impact. **A**: A patient died within 7-day of ICU admission. “SOFA = 16” means that the score of SOFA of the patient was 16 when he admitted to ICU. “meanbp_mean = 57.15” represented that the maximum of mean arterial pressure of the patient was 57.15 mmHg in the first 24 h during ICU stay. “urine output = 34.0” means that the urine output of the patient was 34.0 ml in the first 24 h during ICU stay. **B**: A patient died within 14-day of ICU admission **C**: A patient died within 28-day of ICU admission. “aki_stage_III = 1.0” represented that the AKI stage of the patient was 3 when he admitted to ICU. **D**: A patient surviving after 28-day of ICU admission

Figure 7 illustrates how the SHAP method can be used to explain individual model predictions. Four examples were shown in the figure. It represented an intuitive way to guide the decisions of clinicians and patients and improve their understanding of how the developed model makes a particular prediction. The force plots start at the base value (the average of all predictions). Each predictor (and its corresponding Shapley value) is represented by an arrow which either increases (shown in red) or decreases (shown in blue) the model 's predicted value with respect to the base value. A predictor 's importance is shown by the size of its arrow, where a larger arrow represents a more important predictor. Feature values were listed at the bottom of the plot. Finally, the predicted output value of the model is illustrated by the point where the red and blue arrows meet. Figure 7 (A, B and C) showed the XGBoost model predicted values for three individuals died within the admission of ICU after 7-day, 14-day and 28-day. In Fig. 7 (A), within the first 24 h of ICU admission, the value of urine output of 34.0 ml, the maximum value of International normalized ratio (INR) of 13.6, the maximum value of mean arterial pressure of 57.15 mmHg, age of 91 years old, the SOFA score of 16 when he admitted to ICU, greatly drove to the death of this patient. However, within the first 24 h of ICU admission, the minimum of heart rate (heart_rate_min) of 60.0/min inversely related with predicted death. Contrary to the other three, the patient's ultimate outcome was survival with the output value of -3.30 (Fig. 7 D).

### External validation

**Fig 8:**
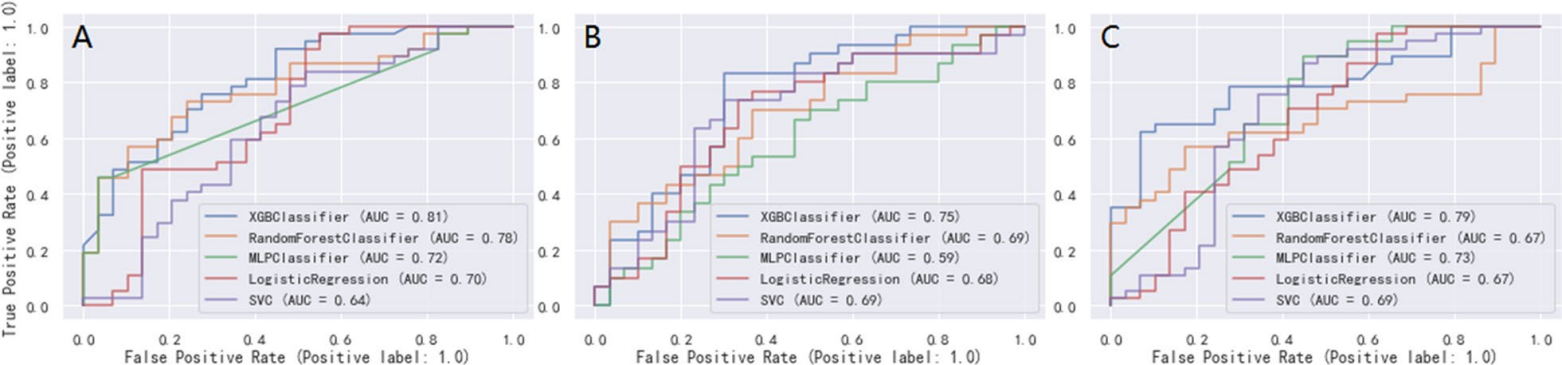
The ROC curves comparison of the five models with the three groups in external validation cohort. blue line = XGBoost model, organ line = random forest classifier model, green line = multilayer perceptron classifier model, red line = logistic regression model, purple line = support vector classifier model; **A**: 7-day group, **B**: 14-day group, **C**: 28-day group

**Fig 9:**
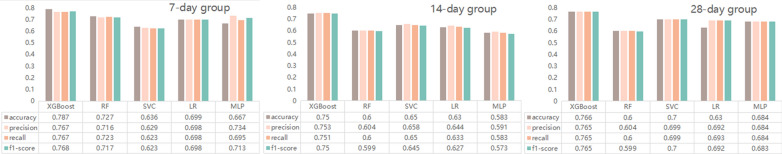
The comparison of performance in the five models with the three groups in external validation cohort

We validated the external cohort enrolled from Hangzhou First People’s Hospital Affiliated to Zhejiang University (Zhejiang, China) between 2018 and 2022. Comparison of variables between the training and external validation cohorts is shown in Additional File3. Patients in the external validation cohort were older than those in the training cohort. Further, there were fewer patients with AKI stage III in the external validation cohort than in the training cohort. Compared with the training cohort, patients in the external validation cohort had lower body weight, higher SOFA scores, and higher mean arterial pressure and mean respiratory rate. In the external validation cohort, the AUCs (95%CI) of 0.70 (0.68, 0.72), 0.78 (0.76, 0.80), 0.81 (0.79, 0.83), 0.72 (0.70, 0.74), 0.64 (0.62, 0.66) in the 7-day group, 0.68 (0.66, 0.70), 0.69 (0.67, 0.71), 0.75 (0.73, 0.77), 0.59 (0.57, 0.61), 0.69 (0.67, 0.71) in the 14-day group, and 0.67 (0.65, 0.69), 0.67 (0.65, 0.69), 0.79 (0.77, 0.81), 0.73 (0.71, 0.75), 0.69 (0.67, 0.71) in the 28-day group were obtained with the LR, RF, XGBoost, MLP, and SVC models, respectively (Fig. 8). XGBoost showed the best performance among all models, especially compared with the SVC and MLP models, respectively. Figure 9 shows the comparison of F1 score, accuracy, precision, and recall among the five models. XGBoost had the best comprehensive performance.

### Models display and application

In order to facilitate the application of our results to clinicians, related researchers, patients and their families, we have developed this prognostic prediction system, which can be assessed at the following websites: https://hanmuya-streamlit-pred-20230419streamlit40-model-tt9kpe.streamlit.app/ [30].

## Discussion

We constructed and validated prediction ML models for prognosis prediction in critically ill patients with S-AKI and improved the interpretability of ML. This study, analyzed 78 features on demographic data, vital signs, and laboratory indicators in the first 24 h after critical care admission; microbiological culture; advanced life support data; and comorbidities using RFE. Forty features were selected to build ML models.

Age at admission; AKI stage III; vital signs within the first 24 h of ICU admission including respiratory rate (mean, min), temperature (mean, min), mean arterial pressure, SpO_2__min and SpO_2__mean, urine output and SOFA score; admitted to ICU, INR; lactate_min and bicarbonate_max of arterial blood; and serum creatinine_max were main variables (for details of selected features, see Fig. 6).

The XGBoost classifier model exhibited the best performance among the five ML classifiers; therefore, this model was used as the baseline model. Meanwhile, using SHAP values and plots, we demonstrated that the ML method could explain key features and establish a high-accuracy mortality prediction model in critically ill patients with S-AKI.The illustration of cumulative domain-specific feature importance and visualized interpretation of feature importance permit physicians to understand the fundamental features of XGBoost intuitively.

This study has made several contributions. First, we introduced the XGBoost algorithm, which has gained popularity in recent years, because of its fast computation, good generalization and high predictive performance [18, 31, 32]. Hyperparameter optimization based on GridSearchCV and SMOTETomek resampling techniques was also used.

Second, the DCA curve was plotted for the clinical application of the XGBoost classifier model and comparison with the other ML models. In the ROC curve comparisons among the five models, XGBoost displayed the best discrimination in the three groups, in the training and external validation cohorts (Figs. 3 and 8). A model is not always clinically useful, even with good discrimination [33]. Clinical intervention guided by the XGBoost model provided a greater net benefit in the training cohort when the threshold probability were 0.2–0.9 (Fig. 5A), 0.3–0.7 (Fig. 5B), and 0.2–0.8 (Fig. 5C). The DCA showed that the XGBoost model had the maximum benefit across the reasonable threshold probabilities, which means the XGBoost model is the optimal and other ML models inferior. In conclusion, the DCA showed that the prognosis prediction model based on XGBoost had a higher clinical application value and better clinical practicability.

Third, one advantage of our study is that we used SHAP values to uncover the black box of ML. The SHAP summary plot illustrated the entire distribution of each feature’s impact on the model output. Sepsis is often accompanied by hypotension and insufficient oxygen supply to the organs. As renal tubules receive a marginal oxygen supply and have high oxygen consumption under physiological circumstances, they are prone to hypoxia and consequent tubular necrosis, which has long been synonymous with AKI [34]. In the 7-day group, SOFA score, admitted to ICU, urine output, meanbp_mean, lactate_min, and SpO_2__mean were 5 of the 10 most important features. These indicators directly or indirectly reflect the hemodynamic status and tissue oxygenation of patients with S-AKI. In the summary plot (Fig. 6A), the deterioration of these indicators greatly contributed to the patient mortality within 7 days of ICU admission.

The composition of the top 20 most influential variables was roughly the same in the summary plot of the three groups and mainly consisted of AKI stage, circulatory status indicators, basic vital signs, age, and weight (Fig. 6 A, B, C). Unexpectedly, the SOFA score was the most important predictor of mortality in S-AKI patients in all three groups; it is a simple but effective rating index to quantify organ impairment by measuring the burden of organ malfunction in severely ill patients, which incorporates measures of cardiovascular, hemostatic, and renal dysfunction [5]. In recent years,the SOFA score has been widely used in clinical practice for sepsis patients. Despite this, the prior models that predicted mortality in S-AKI patients did not use this crucial factor [14, 35].

Force plots visualized individual model prediction as a result of feature contribution. By demonstrating how the XGBoost model generates predictions for four representative individuals, this model provides an intuitive way to guide clinicians’ and patients' decision-making and improves their understanding of how the model makes a particular prediction.

Fourth, in the present study, mortality predictors of S-AKI patients were examined and were found to be consistent with previous findings. Urine output and AKI stage were closely related to renal injury severity [36]. Urine output plays an important role in predicting mortality in S-AKI patients. This result has been confirmed in many related studies. Laranja et al. observed that patients with sepsis-related AKI have lower urine output than those with AKI induced by other factors or with chronic kidney disease [37]. Our findings were consistent with those previous research findings that AKI staging positively correlated with higher mortality [38–41]. These results suggest that AKI occurrence and progress of AKI may lead to blood volume imbalance, fluid electrolyte disturbances, metabolite accumulation, and multiple-organ dysfunction aggravation, forming a vicious cycle in sepsis patients [42, 43].

Elevated lactate levels were closely correlated with poor prognosis in S-AKI patients. Hyperlactatemia was defined as a serum lactate level of > 2 mmol/L, whereas severe hyperlactatemia was defined as a serum lactate level of > 10 mmol/L [44]. In our study, patients in the non-survival group had higher lactate levels than those in the survival group. In other words, the mortality rate of S-AKI patients with severe hyperlactatemia was considerably higher than that of patients with hyperlactatemia. In clinical practice, serum lactate level, as a sensitive indicator to diagnose hypoperfusion or hypoxia, has been shown to correlate with sepsis severity and prognosis [45–47]. Lactate and bicarbonate are both typical metabolic indicators. However, life-threatening S-AKI should not be dismissed in patients with normal lactate levels alone, and those with low bicarbonate levels, regardless of lactate levels, have high mortality rates and should also be considered for early, aggressive therapy [48].

Fifth, our models achieved promising predictive performance and demonstrated robustness and generalizability in the training and external validation cohorts. Predictors included in our models were collected from electronic medical records, and their values were seldom influenced by examiners. Only the most basic and commonly measured clinical data were used in our models, which can improve the generalizability of prediction models in ICUs. Our models were further validated in an external validation cohort that included 100 S-AKI patients from a critical care database. Training cohort data included were from Western countries, but our external validation cohort was from China, demonstrating that the model has applicability in different populations.

Predictors continuously change with changes in the associated pathological phenomenon as the disease progresses. Unlike previous studies that used fixed predictors to predict in-hospital mortality in patients with severe S-AKI [49, 50], we selected different predictors for different survival time of 7, 14, and 28 days, which may represent different stages of the disease, with encouraging results. Rapid disease progression in critically ill patients may cause a delay in prediction using data from 24 h after ICU admission. In clinical practice, there is a growing need for better tools to assess progression and predict earlier which patients need treatment to halt disease progression. With further improvement in the MIMIC database and a better understanding of the pathological mechanism of S-AKI and clinical interventions, combined with interpretable ML algorithms, a prediction system for the prognosis of patients with severe S-AKI at different stages may become a reality.

Our study was not without limitations. First, our training cohort was taken from the MIMIC-IV database, and the majority of the patients were from Western countries, which is quite different from our external validation cohort; second, we did not conduct a more comprehensive study of the database, which may have caused us to overlook some key variables, resulting in potential bias; and third, the retrospective and observational nature of this study may have led to selection bias. Nevertheless, our model still showed satisfactory performance for short-term mortality prediction in the external validation cohort.

## Conclusions

In conclusion, ML methods are reliable tools for the prognosis prediction of patients with S-AKI. Global and local interpretability methods were combined to explain intrinsic information from the XGBoost model, which may prove clinically useful and help clinicians tailor precise management essential to maximize survival in patients with S-AKI.

## Supplementary Information

**Additional file 1:** Missing values information.

**Additional file 2 :**Hyperparameters of ML models.

**Additional file 3: **The comparison of characteristics in training cohort and external validation cohort.

## Data Availability

The datasets used and/or analyzed during the current study are available from the corresponding author on reasonable request.

## Abbreviations

AKI: Acute kidney injury
S-AKI: Sepsis-associated acute kidney injury
MIMIC-IV: Medical information mart for intensive Care IV database
RFE: Recursive feature elimination
SMOTETomek: Synthesizing minority oversampling technology with Tomek link
RF: Random forest
XGBoost: Extreme gradient boosting
MLP: Multilayer perceptron classifier
SVC: Support vector classifier
LR: Logistic regression
ICU: Intensive care unit
ROC: Receiver operating characteristic
AUC: Areas under the ROC curves
RRT: Replacement therapy
IRB: The institutional review boards
MIT: The Massachusetts Institute of Technology
ICD-9: International classification of diseases and ninth revision
SOFA: Sequential organ failure assessment
SCr: Serum creatinine
KDIGO: Kidney disease improving global outcomes
Meanbp: Mean arterial pressure
SpO_2_: Oxyhemoglobin saturation
ABG: Arterial blood gas
INR: International normalized ratio
SHAP: SHapley additive exPlanations
