# Supplementary figures and images for: Construction and validation of prognostic models in critically Ill patients with sepsis-associated acute kidney injury: interpretable machine learning approach
Source: J Transl Med. 2023 Jun 22;21:406. doi: 10.1186/s12967-023-04205-4 (PMC10286378; doi:10.1186/s12967-023-04205-4)

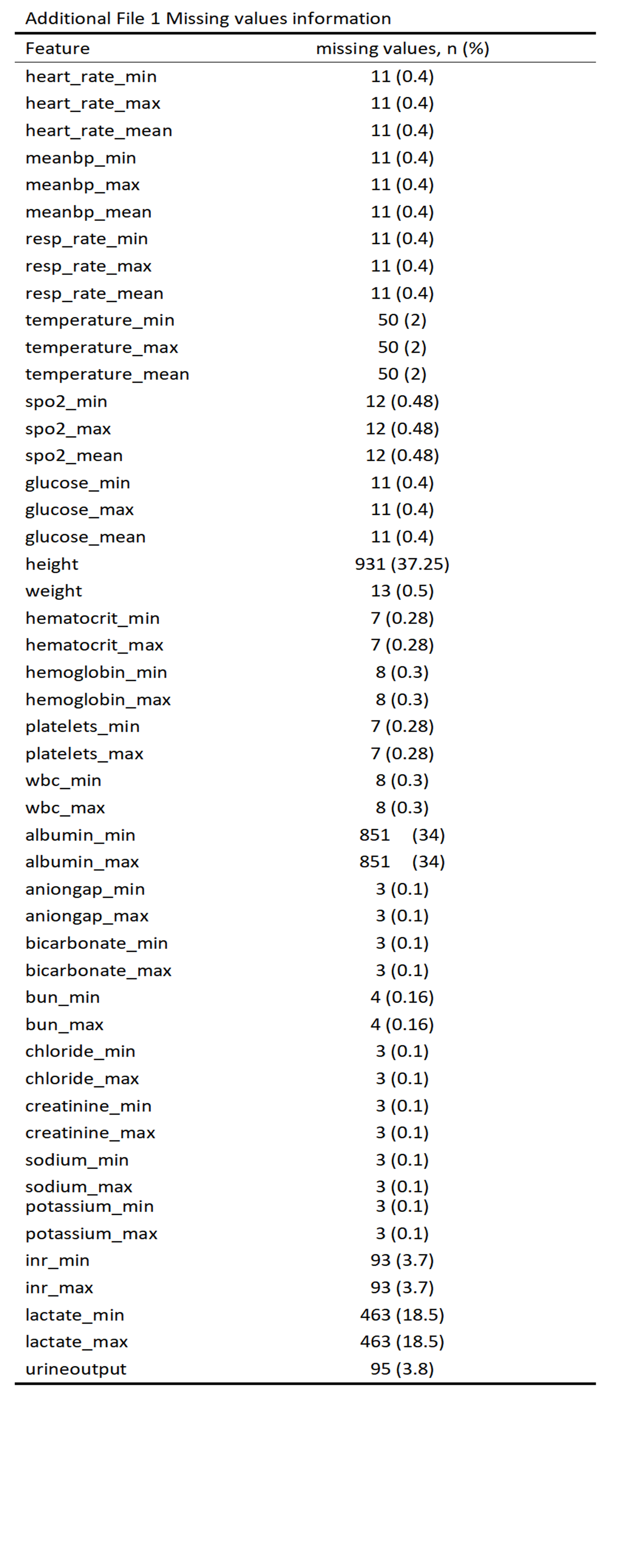

Supplement: Supplementary file 1 — Additional file 1: Missing values information. [file 12967_2023_4205_MOESM1_ESM.png]

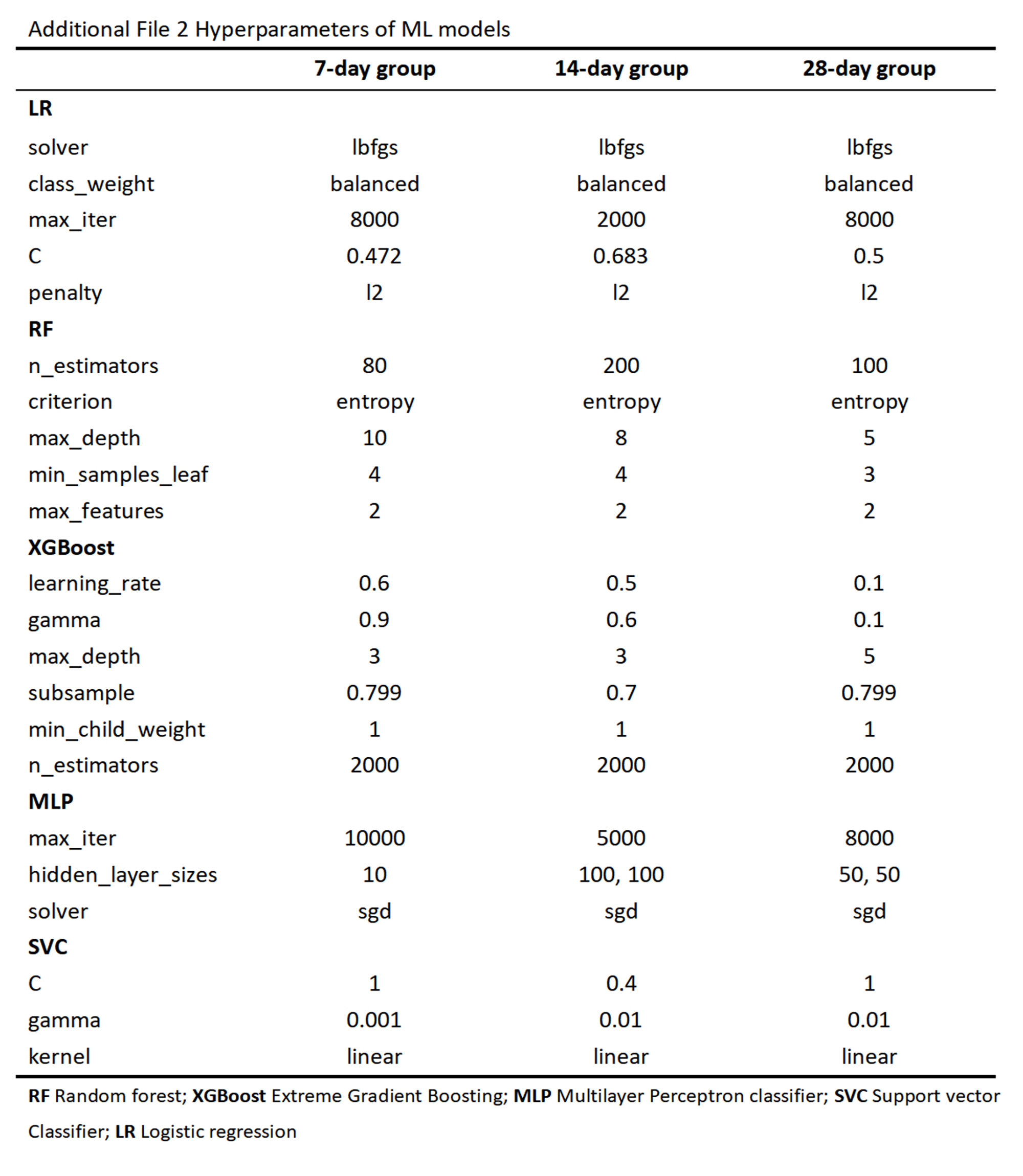

Supplement: Supplementary file 2 — Additional file 2 :Hyperparameters of ML models. [file 12967_2023_4205_MOESM2_ESM.png]

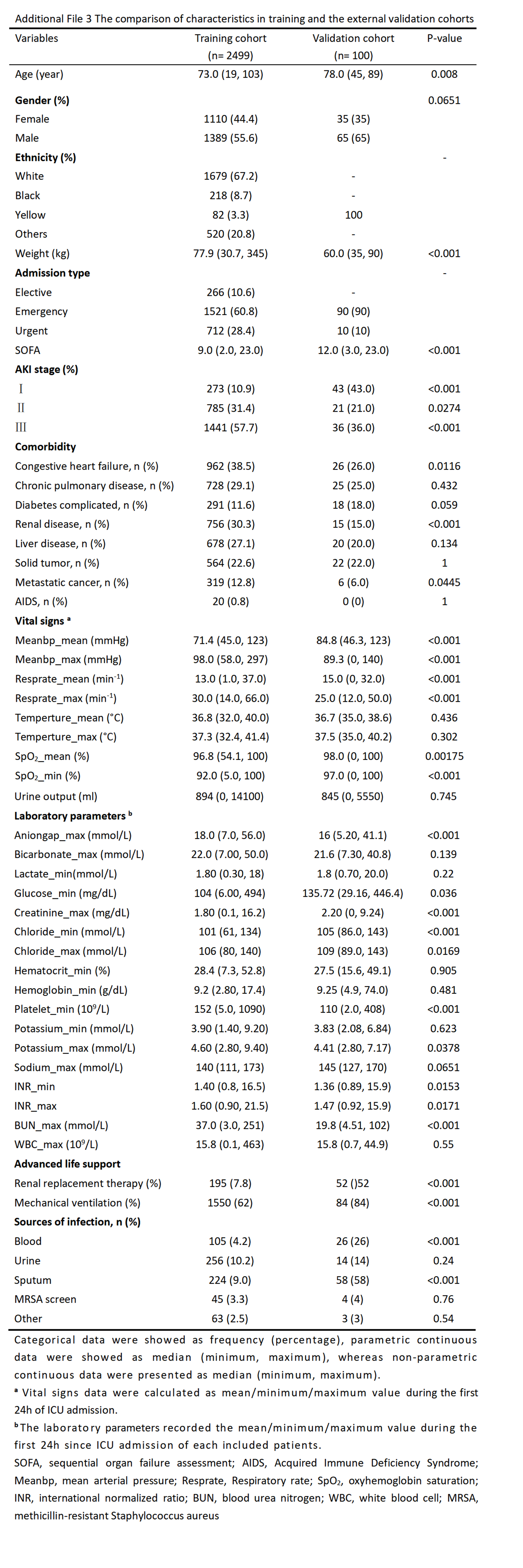

Supplement: Supplementary file 3 — Additional file 3: The comparison of characteristics in training cohort and external validation cohort. [file 12967_2023_4205_MOESM3_ESM.png]
